# Optic nerve crush induces spatial and temporal gene expression patterns in retina and optic nerve of BALB/cJ mice

**DOI:** 10.1186/1750-1326-9-14

**Published:** 2014-04-27

**Authors:** Tasneem P Sharma, Colleen M McDowell, Yang Liu, Alex H Wagner, David Thole, Benjamin P Faga, Robert J Wordinger, Terry A Braun, Abbot F Clark

**Affiliations:** 1 North Texas Eye Research Institute, Ft. Worth, TX USA; 2 Department of Cell Biology & Immunology, NTERI, UNTHSC, Ft. Worth, TX USA; 3 Center for Bioinformatics and Computational Biology, University of Iowa, Iowa, IA USA; 4 Department of Biomedical Engineering, University of Iowa, Iowa, IA USA

**Keywords:** Central nervous system, Optic nerve crush, Retinal ganglion cell, Apoptosis, Axotomy, Neurodegeneration, Regeneration, Microarray, Gene expression

## Abstract

**Background:**

Central nervous system (CNS) trauma and neurodegenerative disorders trigger a cascade of cellular and molecular events resulting in neuronal apoptosis and regenerative failure. The pathogenic mechanisms and gene expression changes associated with these detrimental events can be effectively studied using a rodent optic nerve crush (ONC) model. The purpose of this study was to use a mouse ONC model to: (a) evaluate changes in retina and optic nerve (ON) gene expression, (b) identify neurodegenerative pathogenic pathways and (c) discover potential new therapeutic targets.

**Results:**

Only 54% of total neurons survived in the ganglion cell layer (GCL) 28 days post crush. Using Bayesian Estimation of Temporal Regulation (BETR) gene expression analysis, we identified significantly altered expression of 1,723 and 2,110 genes in the retina and ON, respectively. Meta-analysis of altered gene expression (≥1.5, ≤-1.5, p < 0.05) using Partek and DAVID demonstrated 28 up and 20 down-regulated retinal gene clusters and 57 up and 41 down-regulated optic nerve clusters. Regulated gene clusters included regenerative change, synaptic plasticity, axonogenesis, neuron projection, and neuron differentiation. Expression of selected genes (*Vsnl1*, *Syt1*, *Synpr* and *Nrn1*) from retinal and ON neuronal clusters were quantitatively and qualitatively examined for their relation to axonal neurodegeneration by immunohistochemistry and qRT-PCR.

**Conclusion:**

A number of detrimental gene expression changes occur that contribute to trauma-induced neurodegeneration after injury to ON axons. *Nrn1* (synaptic plasticity gene), *Synpr* and *Syt1 (*synaptic vesicle fusion genes), and *Vsnl1* (neuron differentiation associated gene) were a few of the potentially unique genes identified that were down-regulated spatially and temporally in our rodent ONC model. Bioinformatic meta-analysis identified significant tissue-specific and time-dependent gene clusters associated with regenerative changes, synaptic plasticity, axonogenesis, neuron projection, and neuron differentiation. These ONC induced neuronal loss and regenerative failure associated clusters can be extrapolated to changes occurring in other forms of CNS trauma or in clinical neurodegenerative pathological settings. In conclusion, this study identified potential therapeutic targets to address two key mechanisms of CNS trauma and neurodegeneration: neuronal loss and regenerative failure.

## Background

Central nervous system (CNS) trauma and neurodegenerative disorders trigger a cascade of cellular events resulting in extensive damage to neurons [1-5]. The non-permissive regenerative environment is due to expression of inhibitory cues [3,6-12], glial scarring [5,13], slow clearance of axonal debris [14], and CNS inflammation [15,16]. Regenerative failure is a critical endpoint of these destructive triggers culminating in neuronal apoptosis [3,17,18] and inhibition of functional recovery.

The rodent optic nerve crush (ONC) model is an effective model for CNS trauma and regeneration failure [19-25]. The easy accessibility of the optic nerve (ON), an extension of the CNS, and the reproducibility of the ONC model make it an effective tool to study CNS trauma. Changes in gene expression in rodent ONC models have been previously studied [22,26-30] and include gap associated protein 43 (*Gap43*) [31-33], glial fibrillary acidic protein (*Gfap*) [34-36] and neurofilament deregulation after crush injury [37]. Furthermore, progressive retinal ganglion cell (RGC) degeneration has been associated with loss of trophic support [38,39], stimulation of inflammatory processes/immune regulation [40,41], and apoptotic effectors [39,42-45]. In addition, multiple injury models have been utilized to assess the fate of RGCs after ocular injuries that include ischemia/reperfusion, ON irradiation, ON transections, and traumatic ON injury in rodent and primate models [22,30,46-50].

Although previous studies with CNS trauma models have addressed gene expression changes related to neuronal apoptosis [18,26,39,51], current gaps still exist for identifying long-term neuroprotective and regeneration inducing targets. Additionally, most expression studies for the ONC model have only been performed in the retina or the optic nerve head [3,22,29]. We adopted a distinct strategy from previously published literature by: (a) simultaneously focusing on both the retina and ON, (b) detailing an extended time-course after acute axonal trauma and (c) centering on neurodegeneration and regenerative failure. To pinpoint specific degenerative pathways and identify crucial genes involved with pathological axonal injuries, it is essential to create an extensive molecular gene profile underlying neuronal degeneration and regeneration failure mechanisms. Our study systematically and temporally identified these degenerative mechanisms that ensue after such an insult. To prevent the progression of the disease, new drug therapies geared towards neuroprotection and effective axonal regeneration are required. The purpose of this study was to detect and quantify progressive temporal degenerative changes by: (a) analyzing gene clusters in the retina and ON using Affymetrix microarrays in the neural, immune, and glial cells following ONC and (b) identifying temporal and spatial expression patterns of key gene targets within the retina and ON after trauma. These data will allow the identification of a wide range of potential therapeutic targets associated with neuronal loss and regenerative failure.

## Results

This study highlights common as well as distinct gene expression responses of the retina and ON to ONC injury. To better understand the molecular mechanisms associated with neurodegenerative processes after injury, we first examined the survival of neurons in the ganglion cell layer (GCL) after acute axonal trauma by histological examination of the retinas over an extended 28-day period, which is a well-established time line for RGC death [19]. Second, we identified significant cluster-based changes occurring sequentially in the retina and ON by meta-analysis of the array data. Third, we identified key clusters associated with neuron degeneration to isolate potential underlying damaging gene expression changes occurring within the retina and ON. Lastly, the expression of selective genes was confirmed quantitatively and qualitatively to validate our array data and examine expression of potential therapeutic targets that are affected by CNS trauma.

### Survival of neurons and specificity of gene expression changes following ONC

There was a progressive decrease of neurons in the retinal ganglion cell layer (RGCL) following ONC as assessed by Nissl stained retinal flat mounts (Additional file 1: Figure S1A). Approximately 50% of the cells in the RGCL are RGCs, while the remaining cells consist of displaced amacrine cells, astrocytes, and microglia [19,52-55]. ONC directly damages the ON, eventually leading to the selective death of RGCs. The severity of injury to the RGCs after ONC can vary between studies and depends on: the species and strain of animal used, the quantity of axons affected by the crush, the distance from the globe at which the lesion is performed, the amount of force applied at the site of the lesion, the method used to evaluate damage, and the length of time post crush [37,46,56-58]. A significant sequential decline of RGCL neurons is seen as early as 14 days post crush (dpc) within our model (81.43% ± 16.9% survival, p < 0.01) with increased decline by 21 dpc (58.72% ± 5.70% survival, p < 0.001) and culminating in almost complete loss of RGCs by 28 dpc (54.21% ± 8.27% survival, p < 0.001) (Additional file 1: Figure S1B).

Microarrays were performed following ONC on harvested retina and ON samples from naïve, 3, 7, 14, 21 and 28 dpc mice (n = 5). For the analysis, the retina and ON samples were separately pooled for the experimental and control groups at each time point. Time-course microarray data analysis is challenging in pooled data because each sample has slight variations independent of other samples. These errors can be mitigated to an extent by analyzing the significant temporal changes of genes in pooled samples using the Bayesian estimation of temporal regulation (BETR) analysis [59]. This evaluation allowed us to delineate the differences in percentage of gene changes occurring temporally after ONC within the retinal and ON datasets. BETR probabilities were determined for the total 18,786 genes identified within each dataset. BETR probabilities ranged from 0 to 1 with 0 being the least significantly changed genes temporally and 1 being the most. Genes were then classified into frequency bins based on the range of BETR probabilities.

In the retinal dataset, only 9.17% (1,723 genes out of 18,786 genes) had the highest BETR probabilities within frequency bin 10 (BETR probability - 0.9 to1.0) indicating only a small specific percentage of total genes were altered temporally after ONC trauma (Additional file 2: Figure S2A, Additional file 3: Table S1A). Furthermore, within the ON dataset, only 11.23% (2,110 genes out of 18,786 genes) were in the highest BETR probability range (Additional file 2: Figure S2B, Additional file 3: Table S1B). The small subset of genes identified by BETR analysis correlates with regenerative failure and degeneration that occurs within the retina and ON.

### Cluster specific gene classification following ONC

To extract meaningful biological information from the array data, we used the public data-mining tool Database for Annotation, Visualization and Integrated Discovery (DAVID) to cluster all differentially expressed genes into mechanistic biological categories. Temporal cluster classification is crucial for identifying the neuronal loss mechanisms that are sequentially regulated after trauma. Based on PARTEK fold change levels (≥ 1.5 and ≤ −1.5 compared to the corresponding contralateral control eyes, *q-value* defined by the FDR analogue of the p < 0.05), we temporally categorized the clusters within three gene ontologies (GO); molecular function (MF), biological process (BP) and cellular component (CC) according to the *Mus musculus* genome within the DAVID database.

**Figure 1:**
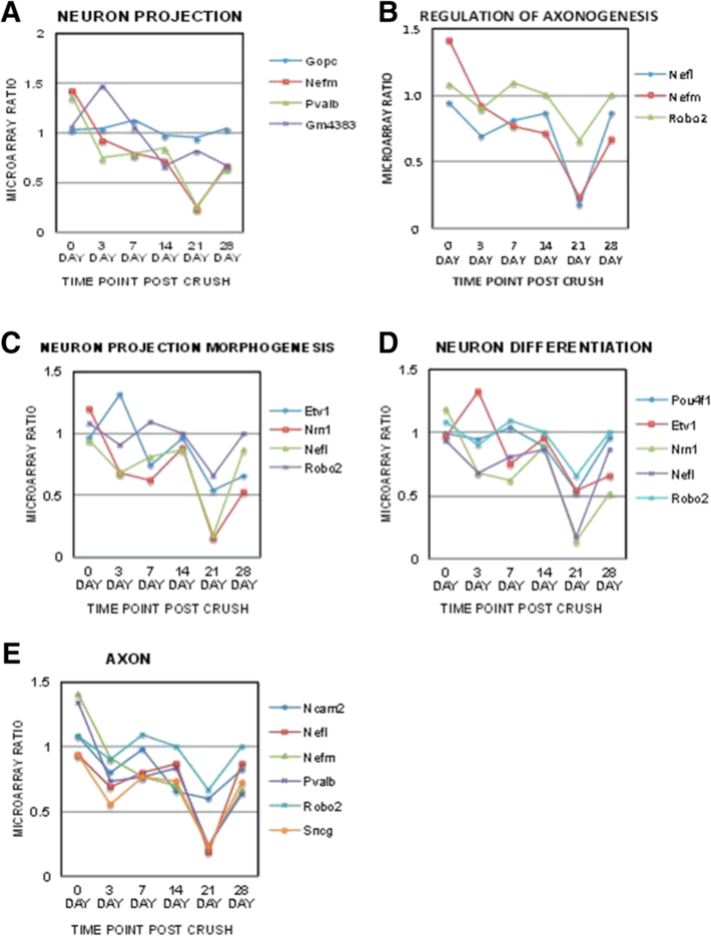
**Temporal changes of specific retinal gene clusters related to neuronal loss and regeneration failure.** Neuron specific and axonal regeneration related neuronal clusters were selected from the retinal GO tables; and the microarray ratios of the genes within each of these clusters were graphed temporally (0 to 28 days post crush (dpc)). Neuronal clusters identified included **(A)** neuron projection, **(B)** regulation of axonogenesis, **(C)** neuron projection morphogenesis, **(D)** neuron differentiation and **(E)** axon. Significance of these clusters was determined using the Benjamini multiple test correction, GO enrichment score *χ*^2^ test and Fishers Exact test (p < 0.05).

**Figure 2:**
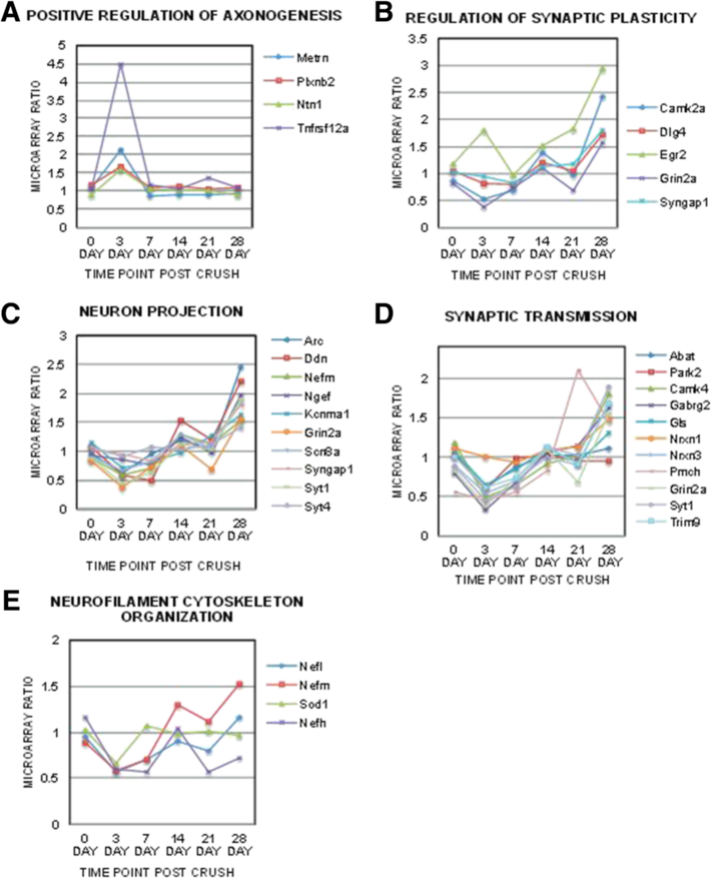
**Temporal changes of specific optic nerve gene clusters related to neuronal loss and regeneration failure.** Neuron specific and axonal regeneration related neuronal clusters were selected from the optic nerve GO tables and the microarray ratios of the genes within each of these clusters graphed temporally (0 to 28 days post crush (dpc)). Neuronal clusters identified included **(A)** positive regulation of axonogenesis, **(B)** regulation of synaptic plasticity, **(C)** neuron projection, **(D)** synaptic transmission, and **(E)** neurofilament cytoskeleton organization. Significance of these clusters was determined using the Benjamini multiple test correction, GO enrichment score *χ*^2^ test and Fishers Exact test (p < 0.05).

A total of 28 up-regulated clusters and 20 down-regulated clusters were significantly identified in the retinal dataset (p < 0.05) and 57 up-regulated clusters and 41 down-regulated clusters were identified within the ON dataset (Tables 1, 2, 3 and 4). To outline neurodegenerative mechanisms, key clusters were identified relating to neuronal loss and regeneration failure from both the retinal (Figure 1) and ON (Figure 2) clusters previously classified in Tables 1, 2, 3 and 4. Each of these key clusters contained a group of genes significantly (p < 0.05) correlating with that specific cluster. The temporal patterns of the microarray gene ratios were graphed according to their association with these clusters for the retina (Figure 1) and ON (Figure 2).

**Table 1:** Temporal classification of up-regulated retinal gene cluster changes following ONC

**Gene ontology**	**Clusters**	**Time point**	**P value**
Molecular function	Structural eye protein	3 dpc	1.90E-06
	Eye development	3 dpc	4.50E-03
	Extracellular matrix binding	3 dpc	5.30E-03
	Calcium ion binding	7 dpc	3.30E-02
	Structural eye lens protein	21 dpc	2.20E-11
	Structural molecular activity	21 dpc	4.60E-06
Biological process	Response to wounding	3 dpc	1.00E-04
	Inflammatory response	3 dpc	3.80E-04
	Defense response	3 dpc	5.90E-04
	Positive regulation of immune system response	3 dpc	1.20E-02
	Rho protein signal transduction	3 dpc	5.90E-03
	Regulation of signal proliferation	3 dpc	2.70E-02
	Defense response	7 dpc	5.40E-04
	Inflammatory response	7 dpc	1.60E-02
	Response to wounding	7 dpc	4.70E-02
	Sensory perception	14 dpc	8.80E-03
	Neurological system process	14 dpc	2.30E-02
	G-protein coupled receptor signaling pathway	14 dpc	3.90E-02
	Macromolecular complex assembly	28 dpc	1.30E-02
	DNA packaging	28 dpc	3.10E-02
	Positive regulation of protein kinase activity	28 dpc	4.50E-02
Cellular component	Extracellular region part	3 dpc	3.20E-03
	Extracellular matrix	3 dpc	7.60E-03
	Lysosome	3 dpc	3.40E-02
	Extracellular region part	7 dpc	3.50E-02
	Microsome	14 dpc	1.70E-02
	Intermediate filament	14 dpc	3.70E-02
	Ribosome	21 dpc	5.00E-03

Gene expression fold-change values were grouped individually from naïve eyes and ONC eyes out to 28 days post crush (dpc). Genes were highlighted based on fold values for up-regulated (≥1.5) retinal datasets. The selected genes were analyzed by gene ontology (GO) based cluster identification at each time point using DAVID. Significance was determined using the Benjamini multiple test correction, GO enrichment score *χ*^2^ test and Fishers Exact test (p < 0.05).

**Table 2:** Temporal classification of down-regulated retinal gene cluster changes following ONC

**Gene ontology**	**Clusters**	**Time point**	**P value**
Molecular function	Structural eye lens protein	7 dpc	3.80E-15
	Structural eye lens protein	28 dpc	2.60E-14
	Pattern binding	28 dpc	3.80E-02
Biological process	Chromatin assembly	7 dpc	4.80E-04
	Regulation of axonogenesis	21 dpc	7.40E-03
	G-protein coupled receptor signaling pathway	21 dpc	2.00E-04
	Neurological system process	21 dpc	7.10E-03
	Intermediate filament bundle process	21 dpc	6.40E-05
	Microtubule based process	21 dpc	5.10E-03
	Axonogenesis	21 dpc	1.70E-02
	Neuron projection morphogenesis	21 dpc	2.00E-02
	Neuron differentiation	21 dpc	4.20E-02
	Cell morphogenesis involved in differentiation	28 dpc	1.20E-02
Cellular component	Nucleosome	7 dpc	7.50E-05
	Neuron projection	21 dpc	5.00E-05
	Axon	21 dpc	6.50E-05
	Neurofilament	21 dpc	1.60E-04
	Intrinsic to membrane	21 dpc	1.30E-02
	Neuron projection	28 dpc	9.90E-03
	Chromosome	28 dpc	3.10E-02

Gene expression fold-change values were grouped individually from naïve eyes and ONC eyes out to 28 days post crush (dpc). Genes were highlighted based on fold values for down-regulated (≤ −1.5) retinal datasets. The selected genes were analyzed by gene ontology (GO) based cluster identification at each time point using DAVID. Significance was determined using the Benjamini multiple test correction, GO enrichment score *χ*^2^ test and Fishers Exact test (p < 0.05).

**Table 3:** Temporal classification of up-regulated ON gene cluster changes following ONC

**Gene ontology**	**Clusters**	**Time point**	**P value**
Molecular function	Chemokine activity	3 dpc	6.50E-06
	Growth factor binding	3 dpc	1.10E-03
	Actin binding	3 dpc	9.10E-03
	Serine type endopeptidase inhibitor activity	7 dpc	7.10E-04
	Chemokine activity	14 dpc	6.70E-09
	Cytokine activity	14 dpc	6.00E-06
	Chemokine activity	21 dpc	1.60E-04
	Cytokine binding	21 dpc	4.30E-04
	Ion channel activity	28 dpc	1.20E-09
	Calcium ion binding	28 dpc	7.70E-07
	GABA receptor activity	28 dpc	4.30E-04
	Neurotransmitter binding	28 dpc	4.00E-03
	Calcium channel activity	28 dpc	9.10E-03
	Protein kinase activator activity	28 dpc	8.80E-03
Biological process	Defense response	3 dpc	6.20E-05
	Translation	3 dpc	1.20E-03
	Cell cycle	3 dpc	3.10E-06
	Leukocyte activation	3 dpc	2.10E-04
	Actin cytoskeleton organization	3 dpc	6.80E-03
	Regulation of adaptive immune response	3 dpc	3.60E-04
	Positive regulation of programmed cell death	3 dpc	1.20E-03
	Positive regulation of axonogenesis	3 dpc	2.10E-02
	Sensory perception	7 dpc	2.90E-02
	Immune response	14 dpc	3.40E-08
	Chemotaxis	14 dpc	2.60E-07
	Response to wounding	14 dpc	1.10E-06
	Cell activation	14 dpc	5.70E-05
	Defense response	21 dpc	1.60E-08
	Response to wounding	21 dpc	4.00E-06
	Chemotaxis	21 dpc	2.60E-06
	Regulation of adaptive immune response	21 dpc	5.40E-06
	Phagocytosis	21 dpc	7.10E-03
	Neuropeptide signaling pathway	21 dpc	2.50E-02
	Ion transport	28 dpc	2.70E-06
	Transmission of nerve impulse	28 dpc	6.30E-08
	Synaptic transmission	28 dpc	1.20E-06
	Synaptic vesicle transport	28 dpc	4.60E-03
	Regulation of synaptic plasticity	28 dpc	2.40E-03
	Regulation of synaptic transmission	28 dpc	6.40E-03
	Synaptogenesis	28 dpc	1.50E-02
	Cell adhesion	28 dpc	3.20E-02
Cellular component	Chromosome	3 dpc	1.50E-09
	Extracellular region part	3 dpc	8.80E-08
	Collagen	3 dpc	6.00E-03
	Focal adhesion	3 dpc	7.20E-03
	Anchoring junction	3 dpc	2.50E-02
	Extracellular region	7 dpc	9.80E-04
	Extracellular region	14 dpc	3.30E-03
	Cell surface	14 dpc	2.80E-04
	Extracellular region	21 dpc	2.60E-04
	Synapse part	28 dpc	1.90E-13
	Postsynaptic membrane	28 dpc	1.90E-07
	Neuron projection	28 dpc	3.30E-07
	Dendrite	28 dpc	2.30E-05
	Synaptosome	28 dpc	3.10E-04
	Postsynaptic density	28 dpc	5.60E-03
	Synaptic vesicle	28 dpc	2.60E-03

Gene expression fold-change values were grouped individually from naïve eyes and ONC eyes out to 28 days post crush (dpc). Genes were highlighted based on fold values for up-regulated (≥1.5) optic nerve datasets. The selected genes were analyzed by gene ontology (GO) based cluster identification at each time point using DAVID. Significance was determined using the Benjamini multiple test correction, GO enrichment score *χ*^2^ test and Fishers Exact test (p < 0.05).

**Table 4:** Temporal classification of down-regulated ON gene cluster changes following ONC

**Gene ontology**	**Clusters**	**Time point**	**P value**
Molecular function	Calmodulin binding	3 dpc	6.50E-04
	Voltage gated ion channel activity	3 dpc	6.70E-04
	Ion binding	3 dpc	3.90E-04
	Enzyme binding	3 dpc	5.80E-03
	GABA receptor activity	3 dpc	6.70E-03
	Ligand gated ion channel activity	3 dpc	1.80E-02
	Calmodulin binding	7 dpc	1.30E-02
	Calcium dependent phospholipid binding	7 dpc	1.40E-04
	Nuclease activity	21 dpc	2.50E-02
	Microtubule binding	21 dpc	2.50E-02
	Motor activity	21 dpc	1.80E-02
	Cytokine activity	28 dpc	2.90E-02
Biological process	Potassium ion transport	3 dpc	4.80E-05
	Cation transport	3 dpc	5.30E-04
	Neurotransmitter transport	3 dpc	3.80E-03
	Synaptic transmission	3 dpc	1.40E-02
	Calcium ion transport	3 dpc	1.10E-02
	Neurofilament cytoskeleton organization	3 dpc	1.20E-03
	Intermediate filament cytoskeleton organization	3 dpc	2.00E-03
	Neurotransmitter transport	3 dpc	3.80E-03
	MAPKKK cascade	3 dpc	2.40E-02
	Microtubule based process	7 dpc	1.10E-02
	Visual perception	14 dpc	4.40E-05
	Cognition	14 dpc	6.50E-03
	Neurological process	14 dpc	1.40E-02
	Microtubule based process	21 dpc	1.30E-05
	Lipid biosynthetic process	21 dpc	2.20E-02
	Visual perception	28 dpc	3.20E-10
	Sensory perception	28 dpc	2.90E-02
	Eye development	28 dpc	4.80E-05
	Immune response	28 dpc	4.70E-02
Cellular component	Synapse	3 dpc	4.30E-06
	Cell junction	3 dpc	6.30E-04
	Post synaptic membrane	3 dpc	1.10E-03
	Presynaptic membrane	3 dpc	5.20E-05
	Synapse part	7 dpc	2.90E-04
	Cell junction	7 dpc	3.90E-03
	Cell projection	7 dpc	3.10E-03
	Clathrin coated vesicle	7 dpc	1.00E-02
	Cytoskeleton	21 dpc	2.30E-02
	Anchored to membrane	28 dpc	4.30E-02

Gene expression fold-change values were grouped individually from naïve eyes and ONC eyes out to 28 days post crush (dpc). Genes were highlighted based on fold values for down-regulated (≤ −1.5) optic nerve datasets. The selected genes were analyzed by gene ontology (GO) based cluster identification at each time point using DAVID. Significance was determined using the Benjamini multiple test correction, GO enrichment score *χ*^2^ test and Fishers Exact test (p < 0.05).

Retinal clusters associated with neuronal loss and regeneration failure included the clusters neuron projection, regulation of axonogenesis, neuron projection morphogenesis, neuron differentiation and axon clusters (Figure 1). Of particular interest was the gene Neuritin 1 *(Nrn1),* which was identified within the neuron projection morphogenesis and neuron differentiation clusters (Figure 1C, D). NRN1 is a secreted GPI-linked protein that stimulates axonal and dendritic arbor growth [60]. Down-regulation of *Nrn1* mRNA expression within the microarray was observed to be biphasic with an initial decline through 7 dpc, a slight increase at 14 dpc and a further decrease by 21 dpc (Figure 1C, D). These biphasic patterns may indicate a transient attempt at neuroprotection/neuroregeneration early in the response to injury.

ON clusters associated with neuronal loss and regeneration identified from the ON cluster tables (Tables 3 and 4) included positive regulation of axonogenesis, regulation of synaptic plasticity, neuron projection, synaptic transmission and neurofilament cytoskeleton organization (Figure 2).

Neuron projection and synaptic transmission clusters both identified key genes called synaptotagmins *(Syt)* that participate in axonal regeneration, including synaptic projection and proper axonal targeting. Expression of *Syt* genes was elevated in the ON at 21 dpc (Figures 2C, D).

By analyzing the retina and ON simultaneously, we were able to observe the temporal response of gene expression in the retina and ON individually as well as in comparison to each other. Neurofilament (NF) genes were identified in most of the retinal and optic nerve clusters. Decreased expression of neurofilament medium (*Nefm*) and light chain (*Nefl*) genes in the retina at 3 and 21 dpc (Figure 1) preceded neuronal loss after axonal damage (Additional file 1: Figure S1B). However, by 28 dpc *Nefm* and *Nefl* expression was elevated in the ON (Figures 2C and E). This pattern of NF expression is consistent with previous studies identifying NF dysregulation during neurodegeneration [61-74].

### Validation of key target genes having differential expression by qRT-PCR

**Figure 3:**
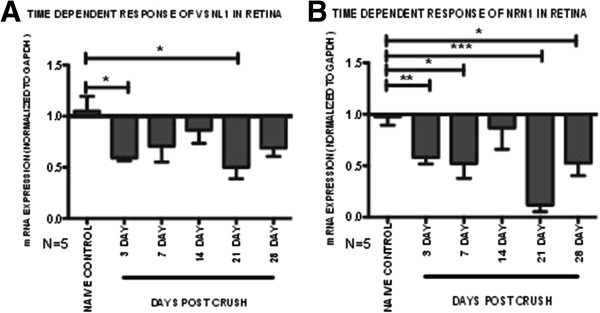
**mRNA expression patterns of selected retinal genes following ONC insult.** Pooled microarray mRNA expression changes were validated in individual samples by qRT-PCR. Relative fold change in each sample was determined based on a 2 fold exponential using mRNA expression values normalized to Gapdh and the contralateral control eye. Fold values of each gene presented as mean ± SEM. **(A)** Visinin like 1 (*Vsnl1)* and **(B)** Neuritin 1 (*Nrn1).* Statistical significance for each time-point determined by one-way ANOVA -Tukey *post hoc* test, * p < 0.05, ** p < 0.01, *** p < 0.001, n = 5.

Analysis of pooled microarray samples does not account for the potential variations that exist between samples and may mask individual sample differences. To confirm individual samples follow the same trend of expression as the microarray data, we used qRT-PCR to determine the expression levels of target genes in each sample. For the retina, we verified two genes (*Nrn1* and *Vsnl1*) that have been previously identified as RGC markers [75,76]. *Nrn1* had similar expression patterns as *Vsnl1* (Figure 3A and B, respectively), and expression of each gene was significantly correlated with the corresponding microarray ratios (*Nrn1* R^2^ = 0.96, *Vsnl1* R^2^ = 0.73) (p < 0.05) (Additional file 4: Tables S2A and B). Both genes displayed a biphasic level of expression with significantly decreased expression from basal naïve levels at 3 and 21 dpc and modestly decreased expression at 14 dpc (p < 0.05, n = 5) (Figure 3).

**Figure 4:**
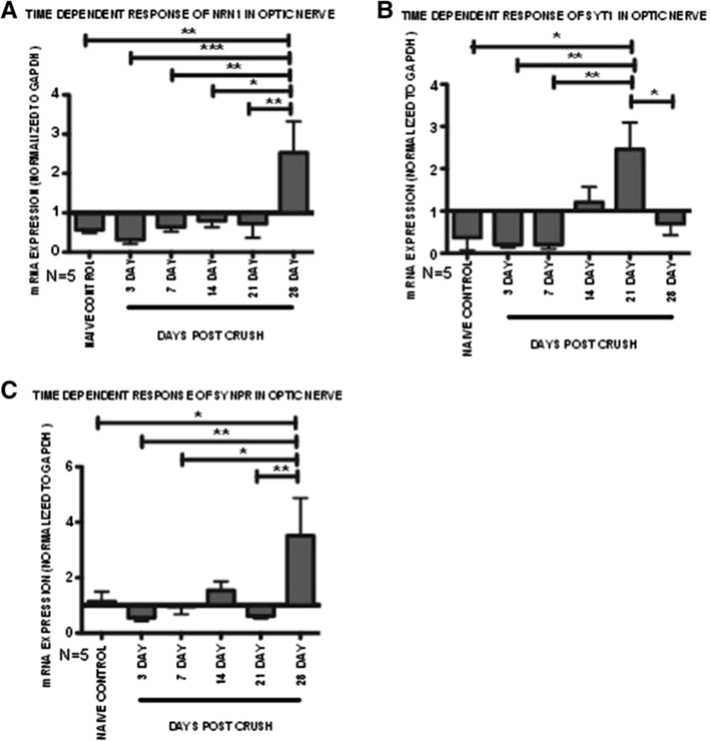
**mRNA expression patterns of selected optic nerve genes following ONC insult.** Pooled microarray mRNA expression changes were validated in individual samples by qRT-PCR. Relative fold change in each sample was determined based on a 2 fold exponential using mRNA expression values normalized to Gapdh and the contralateral control eye. Fold values of each gene presented as mean ± SEM. **(A)** Neuritin 1 (*Nrn1*), **(B)** Synaptotagmin 1 (*Syt1*) and **(C)** Synaptoporin (*Synpr*). Statistical significance for each time-point determined by one-way ANOVA -Tukey *post hoc* test, * p < 0.05, ** p < 0.01, *** p < 0.001, n = 5.

In the ON dataset, *Nrn1*, synaptotagmin 1 (*Syt1*) and synaptoporin (*Synpr)* expression levels were validated by qRT-PCR. We observed significantly increased expression of *Nrn1* at 28 dpc versus all time-points (p < 0.05, n = 5) (Figure 4A). The qRT-PCR results significantly correlate with the microarray data (R^2^ = 0.86, p < 0.05) (Additional file 4: Tables S2A and B). *Syt1* expression was significantly up-regulated at 21 dpc (p < 0.05, n = 5), in contrast to all the other time points (Figure 4B). These results were similar to *Syt1* microarray ratios, in which *Syt1* expression was elevated only at the 28 dpc period. The shift in the time course of gene up-regulation is most likely due to various individual samples that were masked in the pooled microarray samples. Therefore, the linear regression correlation between both sets of data was less than 0.5 (R^2^ = 0.07) (Additional file 4: Tables S2A and B). Increased expression of synaptoporin (*Synpr*) was observed at 28 dpc (Figure 4C) and correlated significantly with gene microarray ratios (R^2^ = 0.71, p < 0.05, n = 5) (Additional file 4: Table S2B). Expression of *Synpr* has been shown in neurons while *Syt1* has been shown in both neurons and is critical in fusion events of astrocytes [77-81]. In addition to synapse formation, *Syt1* has also been shown to regulate the formation of axonal filopodia and branching [80]. The induction of both *Synpr* and *Syt1* expression may be related to synaptic vesicle fusion and release, and the roles of both genes in ONC need to be further explored.

### Immunohistochemical analysis of validated gene targets

**Figure 5:**
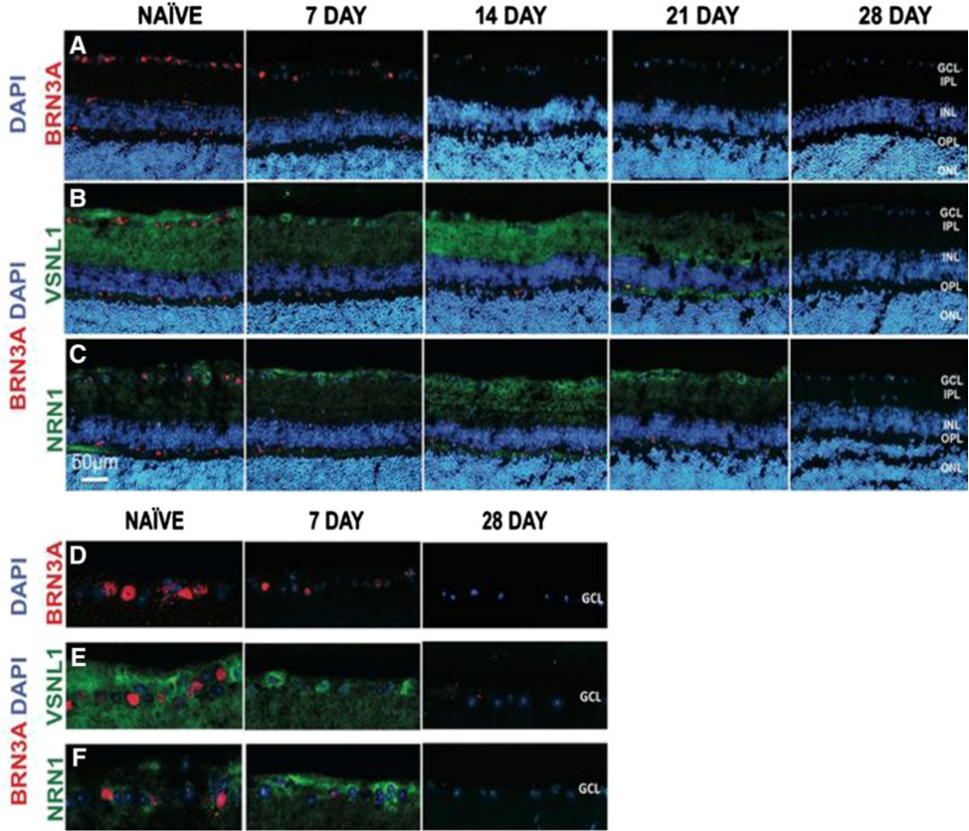
**Expression of Brn3a, Vsnl1 and Nrn1 in the retina following ONC.** Progressive loss of RGCs observed with Brn3a and a biphasic pattern of expression observed for Vsnl1 and Nrn1*.* Time course of retina sections from naïve, 7, 14, 21 and 28 dpc (days post crush) are indicated on the top of the panel. Fluorescence micrographs of retinal tissue sections were immunolabeled with: **(A)** Brn3a (red), **(B)** Vsnl1 (green) and **(C)** Nrn1 (green). Zoomed 50μm length images of the GCL from each of the respective panels above were cropped to show RGC specific staining for naïve, 7 and 28 dpc: **(D)** Brn3a (red), **(E)** Vsnl1 (green) and **(F)** Nrn1 (green). Blue staining indicates DAPI labeled nuclei and all panels show red immunostaining for Brn3a. Scale bar = 50 μm, n = 3. Photomicrographs were captured at 400X original magnification.

Whole retinas were utilized for microarray analysis, potentially masking the changes specific to the RGCs, as they comprise only about 0.5% of the whole retina [82]. To determine temporal protein expression patterns occurring specifically within the RGCs of the GCL, we performed retinal immunostaining. We first tested the expression of Brn3a (brain-specific homeobox/POU domain protein 3A), a well-known marker for RGCs [57,83,84]. As expected, we observed a progressive decrease in Brn3a expression after ONC within the GCL (Figure 5A and D). These results demonstrate a temporal decline in RGCs after axonal injury.

Retinal immunostaining for Vsnl1 and Nrn1 proteins confirmed apparent temporal changes in protein expression after ONC. Within the naïve retinal sections, approximately 50% of the GCL cells were positive for Vsnl1/Brn3a and 47% were positive for Nrn1/Brn3a (Figure 5E and F). A biphasic protein expression pattern was observed for Vsnl1 with decreased expression in the nerve fiber layer (NFL) and inner plexiform layer (IPL) at 7 dpc, increased expression at 14 dpc compared to the naive retina, and a complete loss of expression by 28 dpc (Figure 5B). Focusing on the GCL, the staining pattern also changed at 7 dpc and became more cytoplasmic, in contrast to the diffuse pattern observed in the naïve retinas (Figure 5E). These data verify the *Vsnl1* mRNA expression data (Figure 3A).

A similar biphasic expression pattern was observed for Nrn1 with peak expression at 14 dpc (Figure 5C) and increased nerve fiber layer staining pattern with the GCL at 7 dpc (Figure 5F). Compared to Vsnl1, Nrn1 immunostaining was observed in the ganglion cells and NFL, but not as extensively within the IPL. In addition, previous studies of retinal *Nrn1 in-situ* hybridization exhibited predominant expression within the ganglion cell layer [85], which agrees with our IHC study.

**Figure 6:**
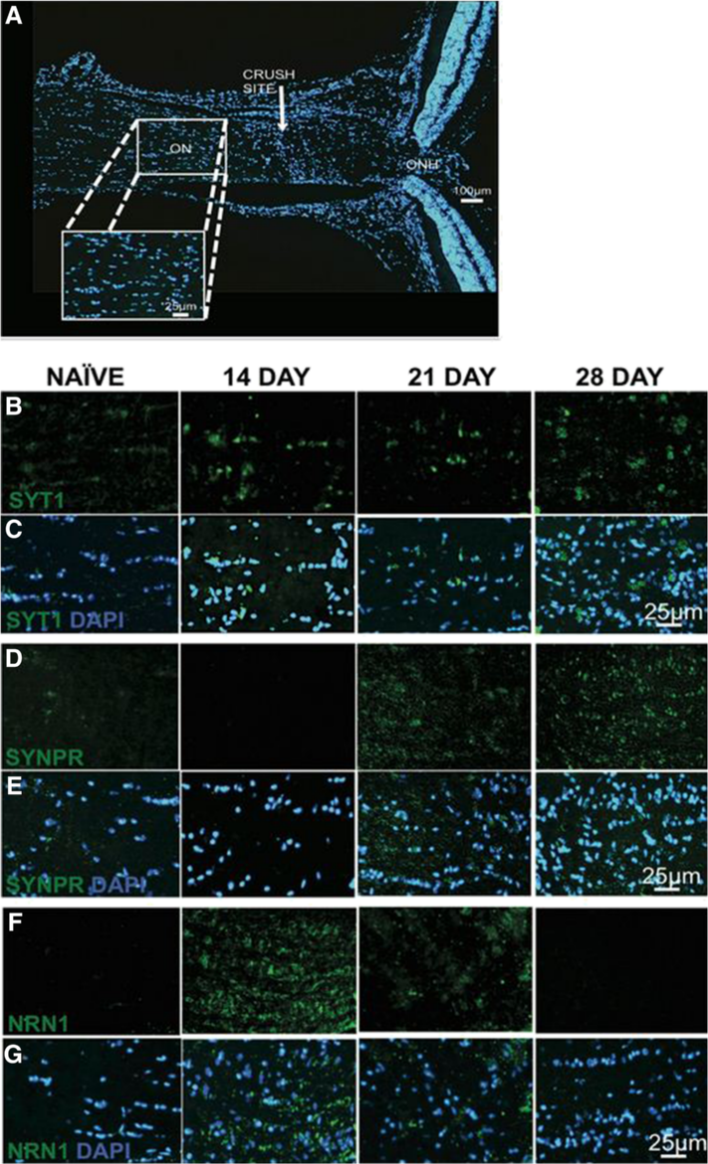
**Expression of Syt1, Synpr and Nrn1 in the optic nerve following ONC.** Differential protein expression of Syt1, Synpr and Nrn1 observed within cytoplasmic region of ON cells following trauma. Optic nerve sections from naïve, 14, 21 and 28 dpc (days post crush) are indicated on the top of the panel. **(A)** Illustrative images captured at each time point for each protein. Fluorescence micrographs of optic nerve tissue sections were captured at 600x magnification and immunolabeled with: **(B)** Syt1 (green), **(C)** Syt1 and DAPI, **(D)** Synpr (green), **(E)** Synpr and DAPI, **(F)** Nrn1 (green) and **(G)** Nrn1 and DAPI. Blue staining indicates DAPI labeled nuclei. (Scale bar = 100 μm (100x), 25 μm (600x), n = 3). Photomicrographs were captured at 600x original magnification for the selected ON genes.

Temporal Syt1, Synpr and Nrn1 protein expression patterns were determined in longitudinal sections of the ON. Images were examined at each time point for each protein as represented by Figure 6A. At 7 dpc, all proteins were individually co-labeled with Nfl to show localization of the axons and the pattern of staining for each protein within the ON (Additional file 5: Figure S3 G-I). The expression pattern of Synpr and Nrn1 was not as intense as Syt1 (Additional file 5: Figures S3 G-I) but still colocalized with Nfl staining. In contrast, the staining pattern of Syt1 colocalized with Nfl and also within the cells surrounding the ON axons (Additional file 5: Figure S3 G). Increased expression of Syt1 was evident at 14, 21 and 28 dpc (Figure 6B and C). Elevated levels of Syt1 were seen within the cytoplasmic region of ON cells through the time course post crush (Figure 6B). Synpr protein expression within the ON was evident at 21 and 28 dpc (Figure 6D and E). Similar to Syt1 expression, Synpr cytoplasmic staining was observed within the ON cells (Figure 6D). The temporal protein expression pattern of Syt1 and Synpr followed the mRNA expression patterns (Figure 4B and C).

In contrast to Syt1 and Synpr, Nrn1 ON protein expression levels (Figure 6F and G) did not match mRNA expression data (Figure 4A). Increased expression of Nrn1 was observed at 14 and 21 dpc (Figure 6F), while increased mRNA expression was observed at 28 dpc (Figure 4A). The offset in the time course for protein expression compared to mRNA expression can be expected due to both mRNA half-life stability and rates of protein synthesis to degradation.

## Discussion

Signaling pathways involved in RGC degeneration are quite complex, and identifying correct target molecules that can mitigate neuronal degeneration and failed regeneration are necessary to develop new neuroprotection strategies. We utilized the ONC mouse model to understand the mechanisms involved in RGC death. ONC directly damages the ON, leading to a progressive loss of RGCs. We identified temporal gene expression changes in the retina and ON after ONC. Key genes associated with neuronal loss and regenerative failure were identified in both retina and ON, and the expression changes were validated by qRT-PCR and immunostaining.

Previously it has been shown that genetic background has an influence on susceptibility to neuronal damage in different inbred mouse lines after neurodegenerative stimuli [20]. C57BL/6 mice are more resistant to ONC, while BALB/c mice are more susceptible to this axonal injury. However, both strains display similar susceptibility to spinal cord injury [20,86]. The differences observed between strains could be partly due to variability in immune response, differences in neuronal stress pathways, and/or activation of alternate cell death pathways [20,87]. In addition, albino rodents are more susceptible to light-induced retinal damage, causing photoreceptor cell death and subsequent retinal degeneration [88,89]. However, retinal degeneration not induced by external factors has not been previously reported in BALB/cJ mice. Thus, our BALB/cJ ONC model is extremely useful for studying the potential mechanisms underlying neuronal cell death due to its susceptibility to crush injury.

Previous ONC studies have observed changes in gene expression within the retina [22,46,90-93] and glial based responses within the ON [29]. Analyzing the retina and ON simultaneously allowed the identification of individual clusters related to neuronal loss and regenerative failure within each tissue separately as well as allowed us to observe the temporal response of gene expression occurring in both the ON and retina with progressive injury to these two tissues.

Neurofilament genes were identified in both the retina and ON datasets. Atypical accumulations of NFs are associated with several neurodegenerative disorders [61-74], and dysregulation of NFs and NF aggregation accompany axonal damage after CNS trauma. NFs have also been associated with CNS diseases and axonal degenerative processes [94]. We show temporal differences in neurofilament expression between the retina and ON suggesting crucial gene changes occur after trauma in the retina and ON. There is progressive decline of retinal *Nfl* expression compared to the elevated expression within the ON out to 28 dpc. These results are consistent with a model in which axonal damage precedes retinal neuronal degeneration and accumulation of damage associated genes occurs within the ON before soma degeneration. The changes in expression patterns identified in our ONC model correlate with previous studies identifying NF dysregulation during neurodegeneration [61-74].

The RGCL comprises multiple cell types including RGCs, amacrine cells, astrocytes, microglia, and vascular cells that interact with the RGC somas. After ONC, these cells also initiate degenerative pathways causing RGC apoptosis [95,96]. Thus, the deregulation of genes observed within the retina is not restricted to RGCs and also represent gene expression of the surrounding cells. Glial fibrillary acidic protein (*Gfap)*, a marker of astrogliosis, is up-regulated after CNS trauma and is used as a universal index of retinal injury [34,96]. *Gfap* is initially up-regulated after ONC [35,36] and showed a similar expression pattern in our retinal dataset. After injury of CNS axons, glial responses around the affected area are increased, and this may contribute to trauma-induced neurodegeneration [97]. By identifying key clusters associated with degeneration of neurons and axonopathy, we were able to isolate potential target genes (*Vsnl1, Nrn1, Syt1, Synpr).*

*Vsnl1* gene is a member of the neuronal subfamily of EF-hand calcium sensor proteins. These proteins play vital roles in cellular signal transduction and neuroprotection/neurotoxicity and have been implicated in neurodegenerative diseases [98,99]. Vsnl1 is predominantly expressed in isolated immuno-panned rat RGCs [75] and has also been shown to specifically label the inner retina (amacrine and RGCs) and the inner plexiform layer of rat, chicken, and bovine retinas [100]. In our study, expression of the *Vsnl1* gene was down-regulated after ONC, which may prevent the survival of RGCs. Although the precise functional roles of *Vsnl1* are still unclear, Vsnl1 proteins may play key roles in membrane trafficking, neuronal signaling, and differentiation [99]. As ON axonal transport is inhibited after ONC, decreased levels of *Vsnl1* may contribute to the deleterious effects on axonal transport mechanisms seen in ONC.

Functionally, Nrn1 acts as a ligand to the insulin receptor [101] and cleavage of the GPI anchor by phospholipase C allows the soluble secreted form to be cell independent [102]. The GPI membrane bound anchor of Nrn1 allows growth promotion as it can stimulate axonal plasticity, dendritic arborization, and synapse maturation in the CNS [60,102]. Conditional knockout of the *Nrn1* gene delays development, maturation of axons and dendritic arbors, synaptic maturation, and effective learning [103]. Neurotrophins such as nerve growth factor (NGF), brain-derived neurotrophic factor (BDNF) and neurotrophin-3 (NT-3) as well as neuronal activity can potentiate the expression of *Nrn1*[104,105]. NGF induces expression of *Nrn1*, which increases neurite outgrowth in a variety of experimental models [104,106,107]. Our studies suggest that after axonal insult, RGCs initially increase *Nrn1* expression for axonal regeneration to overcome obstructed transport mechanisms. These regenerative supportive mechanisms are lost 14 dpc because by then most of the RGCs have been damaged, and the survival of these neurons has progressively decreased. The correlation of retinal protein expression of Nrn1 at 14 dpc mimics the elevated expression of Nrn1 at the same time-point within the ON. Taken together, these data suggest that the dynamic regulation of *Nrn1* may be an effort for axonal regeneration after ONC.

The relative abundance of protein expression may not be proportional to the relative mRNA levels. This lack of correlation in mRNA and protein expression levels could be due to mRNA stability and/or rates of protein synthesis and/or degradation. The slight increase in retinal mRNA expression at 14 dpc (compared to 3 and 7 dpc) is maybe increasing the translation of the Nrn1 within the RGCs soma and Nfl, which is then transported downstream to the ON axons.

The optic nerve includes not only the axons of the RGCs but also astrocytes, microglia, and oligodendrocytes that interact with RGC axons as well as each other [29]. Thus, the expression of genes observed within the ON may represent the beneficial or detrimental effects of neighboring cells surrounding the RGC axons. Differentially regulated genes within the ON expression microarray also identified other key genes associated with synaptic transmission (*Syt1* and *Synpr*) and synaptic plasticity gene (*Nrn1)* that participate in axonal regeneration, including synaptic projection, and proper axonal targeting.

A collection of signaling mechanisms link both axonal tips and dendritic terminals to neuronal soma and nucleus by motor-dependent transport machineries. Signaling complexes could be transported either in endosomes, or as non-endosomal complexes associated with importins and dynein [108]. Essential membrane components of synaptic vesicles and synaptic transmission associated proteins are translated in the soma and get transported to the growing distal ends of extending neurites after crush injury [109,110]. In addition, synaptic vesicles are localized to small vesicles within the neuron, particularly in neuronal axonal processes [111]. Eventually, as axonal transport is inhibited after ONC due to glial scarring [5,13], there is decreased transport of proteins involved in neuroprotection and synaptic plasticity. This causes deleterious effects, eventually leading to decreased synaptic plasticity and transmission at distal ends.

Syt proteins act as synaptic calcium sensors for vesicle fusion in conjunction with SNAREs that facilitate intracellular membrane fusion events [112-114]. Syts have a conserved mechanism of action and are crucial for neuronal Ca^2+^-triggered vesicle fusion [115]. Previous studies have shown *Syt1* to participate in axonal regeneration, including synaptic projection and proper axonal targeting [80,116]. In our study, Syt1 was identified in ON neuron projection and synaptic transmission clusters*.* It appears that the ON attempts to initiate synaptic projection following ONC trauma as shown by the biphasic mRNA and protein expression of Syt1.

Similarly, *Synprs* are essential membrane components of synaptic vesicles [79]. *Synpr* has restricted distribution within the CNS and is present in the telencephalic structures, hippocampus, olfactory bulb, and retina [77,117-119]. *Synpr* plays potential roles in the modulation of synaptic transmission and specificity to neuronal circuitry [79]. Increased protein expression of Synpr in the ON was observed at 21 and 28 dpc. The induction of both Synpr and Syt1 expression may be related to synaptic vesicle fusion and release. After trauma, these synaptic vesicles get transported to the growing distal ends of extending neurites [109,110]. Eventually, as the RGCs are trying to overcome regenerative failure, they may increase expression of Syt proteins within their axons in attempt to induce synaptic plasticity and transmission at distal ends. Elevated expression of Synpr and Nrn1 suggests they are mediating synaptic differentiation as synaptic organizing proteins, but the deregulation of mRNA expression and eventually protein expression may be a futile attempt at ON regeneration in late pathogenesis.

We have explored temporal gene expression changes after ONC axonal injury that can be extrapolated to other CNS traumas. Although there are gene expression differences between the retina and brain, similar differences also occur within discrete regions of the brain as each part of the brain has different motoric, sensory, and cognitive functions. For example, gene expression in the cerebellum differs the most from the other regions of the brain [120,121] and has also been reported in inbred strains of mouse brain [122]. In addition, inter-individual differences have also been reported within a species [121]. As is the case while studying any trauma or disease model, only a generic evaluation can be made in terms of relevance to other regions in the CNS. In conclusion, the ONC model has identified two key mechanisms of CNS trauma and neurodegeneration: neuronal loss and regenerative failure. Dysregulation of *Vsnl1*, *Syt1*, *Synpr* and *Nrn1* gene expression may play an important role in neurodegeneration and potentially provide unique targets for intervention.

## Conclusions

The current study delineates the gene expression profile associated with neurodegeneration and regenerative failure after ONC-induced CNS trauma. CNS trauma causes degeneration of neurons and axonopathy, which is evident in neurodegenerative diseases such as Parkinson’s, Alzheimer’s, and glaucoma [1-5]. The susceptibility of the neurons to acute axonal injury allowed the identification of gene expression changes that occur before neuronal loss. Using the reproducible ONC model of CNS trauma, we were able to: (a) examine gene expression changes within the retina and ON, and (b) visualize protein expression patterns of key selected genes associated with neuron loss and regenerative failure within the retina and ON after ONC. BETR analysis of microarray gene expression data was utilized to show that a select small subset of genes was affected at multiple time points following ONC. Bioinformatic meta-analysis identified gene clusters associated with regenerative changes, synaptic plasticity, axonogenesis, neuron projection, and neurodegeneration. A neurite synaptic plasticity gene (*Nrn1*), synaptic vesicle fusion genes (*Synpr* and *Syt1*), and neuron differentiation associated gene (*Vsnl1*) were a few of the key temporally regulated genes identified in our study. In conclusion, analysis of these gene arrays and protein expression patterns allowed the detection, quantification and visualization of key differentially regulated genes after ONC. This study has identified potential pathogenic genes and possible new therapeutic targets to address two key mechanisms of CNS trauma: neuronal loss and regenerative failure.

## Methods

### Animals

BALB/cJ mice aged 2–4 months were utilized for all the experiments and were obtained from the Jackson Laboratories (Bar Harbor, ME). The mice were housed and maintained in a 12-hour light/dark cycle and fed ad libitum. All procedures were performed in accordance with Association for Research in Vision and Ophthalmology Statement on the Use of Animals in Ophthalmic and Vision Research and the University of North Texas Health Science Center (UNTHSC) Institutional Animal Care and Use Committee regulations (IACUC protocol # 2011/2012-58-A04, approved October 8th 2012).

Mice were divided into three separate groups according to the experiment. The time course of the ONC included six different time-points (naïve (0), 3, 7, 14, 21 and 28 dpc). To determine the percentage of neurons surviving after crush, 8–9 mice per time-point were used for retinal Nissl staining. For the microarray studies and qPCR validation, 5 mice were used per time-point. After the retinal and ON tissues were harvested, the samples were divided into two parts. cDNA was made from half of each individual sample for qPCR validation, while the remaining portion of samples were pooled to generate one sample for each experimental time point. Microarray analysis was performed on control and ONC retina and ON samples for each time point. To qualitatively identify protein expression, three mice per time-point were utilized for IHC.

### Optic nerve crush model

The ON of the left eye was crushed 0.5 mm posterior from the globe for 4 seconds using the Nickell’s technique [19]. Briefly, mice were anesthetized by intraperitoneal injection of ketamine (100 mg/kg) and xylazine (10 mg/kg) and an incision was made along the superior orbital margin. The ON (left) was exposed and crushed using a self-closing jeweler’s forceps to ensure reproducibility and constant force. Extreme care was taken not to damage the ocular blood vessels. Indirect ophthalmoscopy was performed to ensure retinal circulation was not blocked. The contralateral eye was used as the uncrushed control.

### Characterization of optic nerve crush model

To quantify cell loss from the retinal RGCL, retinas from fixed eyes were dissected, flat mounted and Nissl stained with cresyl violet stain as previously described [19,87,123]. Eyes were fixed in 4% paraformaldehyde in phosphate buffered saline (PBS; 0.1M phosphate and 100mM NaCl buffer (pH 7.4)) for an hour at room temperature. After fixation, the eyes were rinsed with PBS, and the posterior cup isolated and placed in 0.3% Triton-X 100 PBS for 16 hours at 22°C. The tissues were then placed in 3% H_2_O_2_ and NaH_2_PO_4_ overnight. The retinas were dissected, cut into four quadrants, and mounted RGC side up on positively charged glass slides (Fisher Scientific, Chicago, IL). The slides were then air dried and flattened with coverslips using 10 g weights. The dried retinas were stained with 1% cresyl violet acetate in 0.25% acetate for 30–45 seconds. After staining, the retinas were dehydrated in 90% and 100% ethanol and cleared with xylene to reduce background staining and mounted with a coverslip.

To determine the density of remaining RGCL neurons within each retina, two digital images from each quadrant (peripheral and mid-peripheral region - four quadrants/retina) were captured at 400 X magnification. A total of 8 images per retina were counted using Adobe Photoshop software. Cell counts were analyzed by comparing the experimental retinas against the contralateral control retinas (cell counts ± SD) at each time point. Quantification of percentage neuron survival following ONC from 3 to 28 dpc was performed. Data points (Additional file 1: Figure S1 B) represent mean ± SD of surviving neurons after crush normalized to contralateral control eyes. Statistics were determined using one-way ANOVA -Tukey *post hoc* test, ** p < 0.01, *** p < 0.001, n = 8-9 eyes/time-point.

### RNA processing

Fresh retina and ON samples (from the globe to the chiasm) were cleanly dissected without any contamination from surrounding tissue. In brief, after euthanization each globe was harvested from the mouse eye socket at the globe and optic nerve head (ONH) juncture. The globe was transferred to a clean petri dish and opened along the limbus. The retina was harvested from the posterior cup and the ONH removed using a trephine. For the ON, the skull was opened and each left and right ON between the globe and chiasm was harvested separately. All samples were collected in 1 ml of TRIzol (Invitrogen, Grand Island, NY) and homogenized using 5 mm steel beads in the TissueLyser LT (Qiagen, Valencia, CA) for five minutes at 50 oscillations/second. For phase separation 50 μl of BAN Phase Separation Reagent (Molecular Research Center, Cincinnati, Ohio) was added to the homogenized samples, and samples were centrifuged at 14,000 rpm for 15 minutes. The upper aqueous phase was transferred to an RNeasy mini column (Qiagen, Valencia, CA) and processed according to manufacturer’s protocol. The total RNA was re-suspended in 20 μl of nuclease-free water and quantified using the Thermo Scientific NanoDrop 2000 (NanoDrop products, Wilmington, DE). Integrity of the RNA was measured by calculating the RNA integrity number (RIN) using the Agilent Bioanalyzer (Agilent Technologies, Santa Clara, CA), and samples with RIN values greater than 7 were used for microarray analysis.

### Affymetrix gene chip arrays

For microarray analysis, 420 ng of RNA from each retina sample and 100 ng from each ON sample was pooled to a total of 2100 ng and 500 ng, respectively, and this was performed for each experimental and control group at each time point. Microarray hybridizations were performed at the University of Iowa DNA Core Facility. Total RNA (50 ng) was converted to SPIA (Single Primer Isothermal Amplification) amplified cDNA using the WT-Ovation Pico RNA Amplification System, v2 (NuGEN Technologies, San Carlos, CA). The amplified SPIA cDNA product was purified through a QIAGEN QIAquick PCR Purification column (QIAGEN). Five micrograms of this product were fragmented (average fragment size = 85 bases) and biotin labeled using the NuGEN FL-Ovation cDNA Biotin Module (NuGEN Technologies). The resulting biotin-labeled cDNA was mixed with Affymetrix eukaryotic hybridization buffer (Affymetrix, Inc., Santa Clara, CA), placed onto Affymetrix Mouse Gene 1.0 ST arrays and incubated at 45°C for 18 h with 60 rpm rotation in an Affymetrix Model 640 Genechip Hybridization Oven. Following hybridization, the arrays were washed, stained with streptavidin-phycoerythrin (Molecular Probes, Inc., Eugene, OR), and the signals were amplified with an anti-streptavidin antibody (Vector Laboratories, Inc., Burlingame, CA) using the Affymetrix Model 450 Fluidics Station. Arrays were scanned with the Affymetrix Model 3000 scanner with 7G upgrade, and data were collected using the using the GeneChip operating software (GCOS) v1.4.

### Bioinformatic analysis of gene expression datasets

Microarray data were imported into the Partek Genomics Suite 6.6 software (Partek Inc., Louis, MO) and normalized based on the robust multi-array average (RMA). To further confirm the purity of each extracted tissue, we examined the expression of retina specific genes in the ON tissue and ON genes in the retina tissue. There was greater expression of retina specific markers: *Rho*, *Nr2e3*, *Nrl* and *Crx* in the retinal samples, while these genes were at the lower limits of detection in the ON samples. Conversely, there was greater expression of myelin marker genes *Mag*, *Mobp*, *Mog*, *Mbp* and *Plp1* in the ON samples compared to the retina samples. In addition, we tested expression levels of *Rho* and *Mbp* by qPCR in both tissues (Additional file 6: Figure S4).

For the microarray analysis, the ONC samples were compared to the control samples and the microarray ratios and log_2_ fold values calculated at each time point. Up and down-regulated genes were identified for both datasets (retina and ON) with a selective filter of ≥1.5 and ≤ −1.5 fold values. The fold values were based on the *q-value* defined by the FDR analogue of the p <0.05. The genes were further analyzed using the publicly available bioinformatics software Database for Annotation, Visualization and Integrated Discovery (DAVID). Gene ontology (GO) based cluster analysis was performed to identify possible enrichment of genes (GO enrichment score calculated using a *χ*^2^ test) using differentially regulated genes per time point. The Fishers Exact p value is calculated by DAVID to identify GO enrichment based clusters and any p < 0.05 were considered to be significant based on the Benjamini multiple test correction and were enriched in the annotation category [124,125]. Neuronal clusters were identified at specific time points and their genes graphed temporally under each GO category.

### Identification of specific gene expression changes following ONC by Bayesian estimation of temporal regulation analysis

Analysis of time-course microarray data was performed using Bayesian Estimation of Temporal Regulation (BETR) analysis to account for any variations between individual samples within the pooled samples. The BETR expression probabilities were estimated using Probe Logarithmic Intensity Error with GC-background correction, a routine built into the Affymetrix Power Tools toolkit. Expression estimates for 11 housekeeping genes across all time-points were used to create a linear model between the average expression level and variance of each gene and housekeeping genes. This model was used to simulate additional readings for all estimated transcripts at each time point, which were subsequently used as additional inputs to the BETR [59] R package. From this algorithm output final BETR probabilities were determined for the total 18,786 genes identified within each of the retina and ON gene expression datasets (Additional file 3: Table S1 A, B). BETR probabilities ranged from 0 to 1 with 0 being the least significantly changed genes temporally and 1 being the most. Genes were then classified into frequency bins based on the range of BETR probabilities. Each frequency bin identified a range of 0.1 differences in BETR probabilities and bins ranged from the lowest 1 (BETR probability range of 0–0.1) to frequency bin 10 (BETR probability range of 0.9-1) (Additional file 3: Table S1A, B). We considered low BETR probabilities of frequency bins <5 to reflect no significant changes in gene expression, while high BETR probabilities (0.9-1.0) within frequency bin 10 to represent significant changes in gene expression over time.

### Microarray confirmation through real-time qRT-PCR

Quantitative real-time PCR (qPCR) was used to validate the temporal gene microarray expression ratios for the differentially expressed genes. From the retinal data sets, two genes (*Vsnl1* and *Nrn1*) were selected, while from the optic nerve data set, three genes were chosen (*Syt1, Synpr* and *Nrn1*) (Additional file 4: Table S2A). Reverse transcription was performed using the iScript™ cDNA synthesis kit (Bio-Rad Laboratories, Hercules, CA). Each sample (500 ng of RNA) was reverse transcribed as per manufacturer’s protocol. Gene specific primers were designed (MGI database) (Additional file 7: Table S3) and PCR products sequenced to confirm the specificity of each primer’s transcript (Genewiz Inc, NJ). qPCR was then performed in the BioRad CFX96 real time system (Bio-Rad Laboratories, Hercules, CA) using the SSoAdvanced™ SYBR Green master mix (Bio-Rad Laboratories, Hercules, CA). Cycles for the qRT-PCR were run as described in Additional file 8: Table S4. The cycle threshold (C_t_) was assigned as log_2_ of PCR amplification. Technical duplicates for each sample were averaged, and each ONC and control samples were normalized to their own *Gapdh* C_t_ values. The difference between the ONC sample (experimental) and control sample ΔC_t_ values was used to determine the relative fold change in each sample based on a 2 fold exponential. Control qRT-PCR reactions were performed in the absence of a cDNA template. Gene expression fold changes were graphed temporally for each dataset and compared to temporal microarray ratios from the Partek analysis. Statistical analysis for qPCR was performed using GraphPad Prism Software (Mean ± SEM) using one-way ANOVA (Tukey *post hoc* test) with a p < 0.05 considered statistically significant. Regression analysis was performed between qRT-PCR and microarray ratios, and the R^2^ coefficient of determination calculated and p < 0.05 were considered statistically significant (Additional file 4: Table S2 B).

### Immunohistochemistry

IHC was performed to validate protein expression of qRT-PCR confirmed genes and to localize target proteins in the retina and ON. Whole eyes were harvested and fixed in 4% paraformaldehyde for 2 hours at room temperature. After fixation, the tissue was placed in 20% sucrose overnight at 4°C and embedded in optical cutting temperature (OCT) the next day. Sections (10 μm) were cut using a cryostat (Leica Biosystems - Richmond, IL). Cross sections of retina were transferred to Superfrost glass slides (Fisher Scientific - Chicago, IL). Slides were incubated in PBS for 10 minutes and blocked with SuperBlock™ Blocking Buffer (Fisher Scientific, Chicago, IL) at room temperature for one hour. Primary antibodies (Additional file 9: Table S5) were diluted in Superblock™. Each slide was probed with the respective primary antibody and incubated overnight at 4°C. Sections were then washed 3 times with PBS for 10 minutes each and incubated with Alexa Fluor secondary antibody (Additional file 9: Table S5) for 1 hour at room temperature. Slides were rinsed three times with PBS and mounted with ProLong® Gold anti-fade reagent with DAPI (Molecular Probes, Grand Island, NY). Sections were observed and captured using a Nikon Eclipse Ti-U Microscope (Nikon, Melville, NY) containing the Nuance Multispectral imaging system and analyzed using Adobe Photoshop CS5 software. Negative control images of retina and ON sections with no primary antibody are presented in Additional file 5: Figures S3 A-F.

### Availability of supporting data

GEO Accession Number: Series GSE44708.

## Abbreviations

CNS: Central nervous system; SCI: Spinal cord injury; ONC: Optic nerve crush; RGC: Retinal ganglion cells; BETR: Bayesian Estimation of Temporal Regulation; DAVID: Database for Annotation, Visualization, and Integrated Discovery; MF: Molecular function; BP: Biological process; CC: Cellular component; NFL: Nerve fiber layer; GCL: Ganglion cell layer; DPC: Days post crush; OCT: Optical cutting temperature; PBS: Phosphate buffer saline solution; IHC: Immunohistochemistry; ANOVA: Analysis of variance; DAPI: 4′,6-diamidino-2-phenylindole; qRT-PCR: Quantitative real time polymerase chain reaction; NCBI: National center for biotechnology information; GO: Gene ontology; RIN: RNA integrity number

## Competing interests

The authors declare that they have no competing interests.

## Authors’ contributions

TPS performed the RNA extraction, tissue sample staining, bioinformatics Partek and DAVID analysis, qRT-PCR and correlation analysis of all optic nerve crush samples; including writing all sections of the manuscript. CM and YL both performed the optic nerve crush and subsequently CM did the Nissl stained retinal flat mount neuron survival analysis while YL prepared the immunohistochemistry sections. BF processed image data. DT performed differential expression analysis and AW analyzed the microarray data using the BETR analysis. AFC, RJW and TAB conceived the study, actively participated in the design and coordination of the study, reviewed all the data, and helped draft the manuscript. All authors read and approved the final manuscript.

## Supplementary Material

Additional file 1: Figure S1 A, B: Optic nerve crush (ONC) significantly reduces neurons in the retinal ganglion cell layer (RGCL). Click here for file

Additional file 2: Figure S2 A, B: Frequency distribution of genes altered following optic nerve crush. Click here for file

Additional file 3: Table S1 A, B: BETR probabilities based retinal and ON genes distributed within frequency bins. Click here for file

Additional file 4: Table S2 A, B: Microarray ratios and linear regression correlation values of selected target genes. Click here for file

Additional file 5: Figure S3 A-I: Naïve control images and expression of Syt1, Synpr, Nrn1 and Nfl in the ON at 7 days post crush. Click here for file

Additional file 6: Figure S4 A, B: Expression of tissue specific genes within normal retina and ON samples. Click here for file

Additional file 7: Table S3 Primers for key genes validated from the retina and optic nerve datasets. Click here for file

Additional file 8: Table S4 qRT-PCR cycles performed for confirming retina and optic nerve dataset gene expression levels. Click here for file

Additional file 9: Table S5 Antibodies against key proteins validated by IHC. Click here for file
