# Supplementary figures and images for: Optic nerve crush induces spatial and temporal gene expression patterns in retina and optic nerve of BALB/cJ mice
Source: Mol Neurodegener. 2014 Apr 27;9:14. doi: 10.1186/1750-1326-9-14 (PMC4113182; doi:10.1186/1750-1326-9-14)

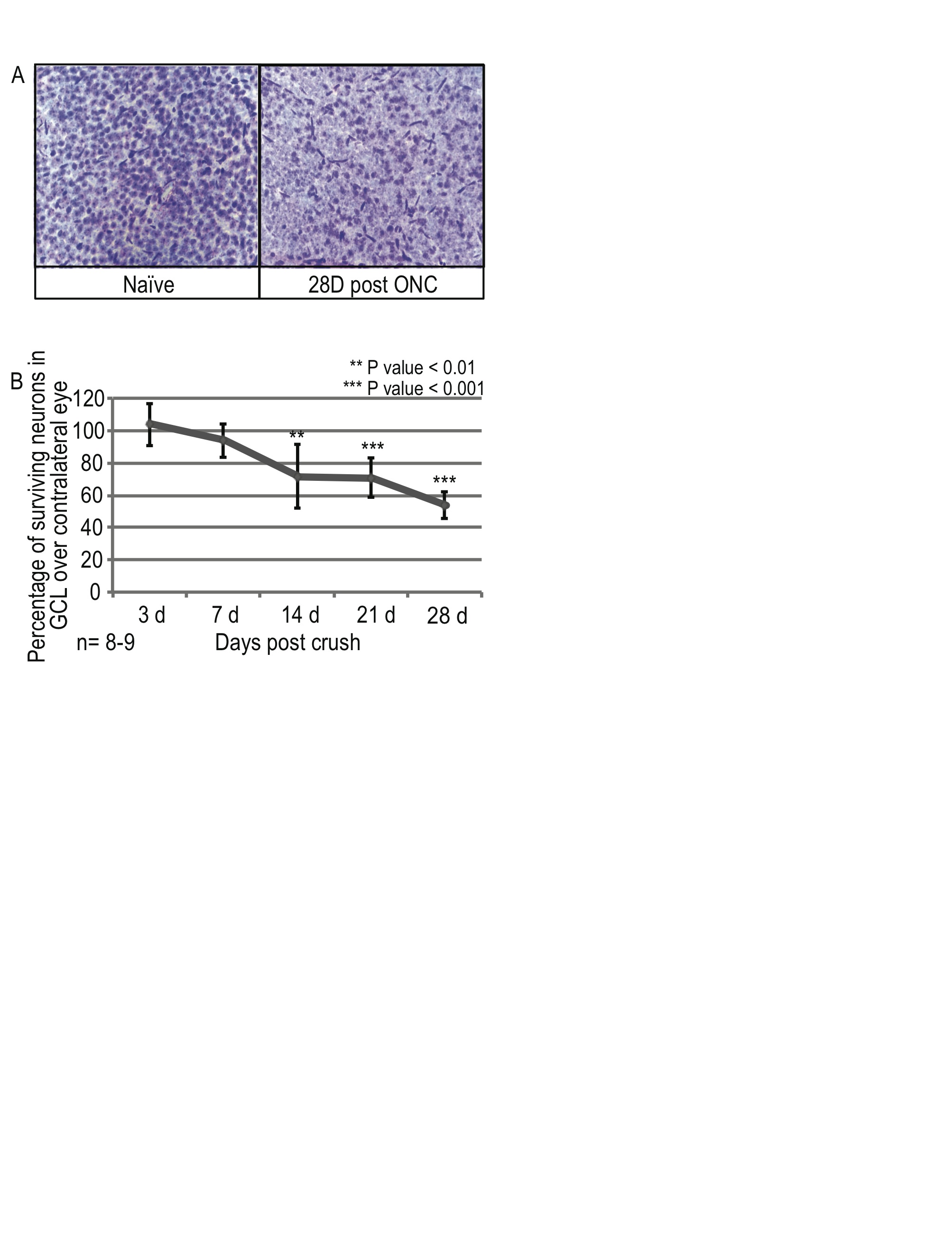

Supplement: Additional file 1: Figure S1 — A, B: Optic nerve crush (ONC) significantly reduces neurons in the retinal ganglion cell layer (RGCL). [file 1750-1326-9-14-S1.jpeg]

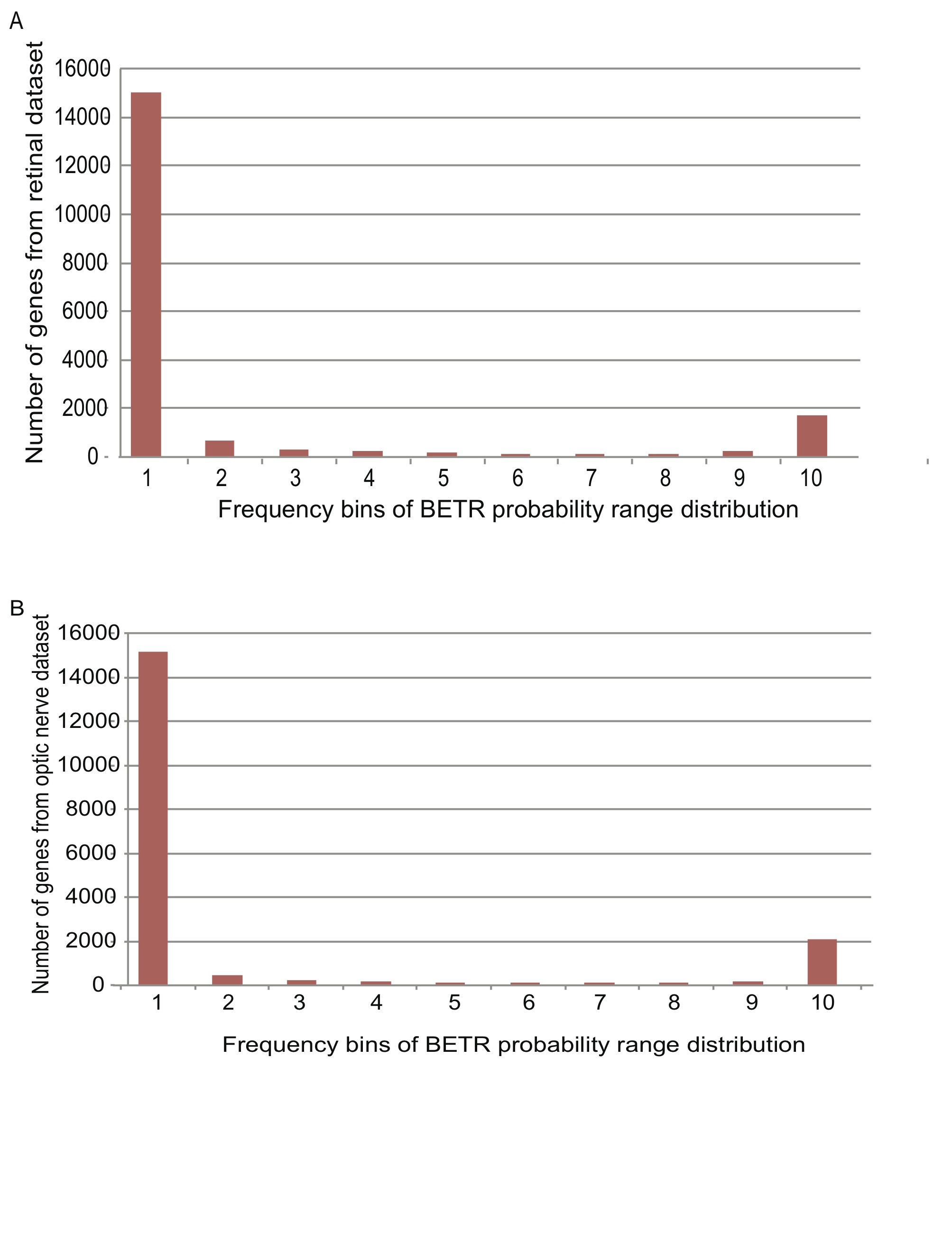

Supplement: Additional file 2: Figure S2 — A, B: Frequency distribution of genes altered following optic nerve crush. [file 1750-1326-9-14-S2.jpeg]

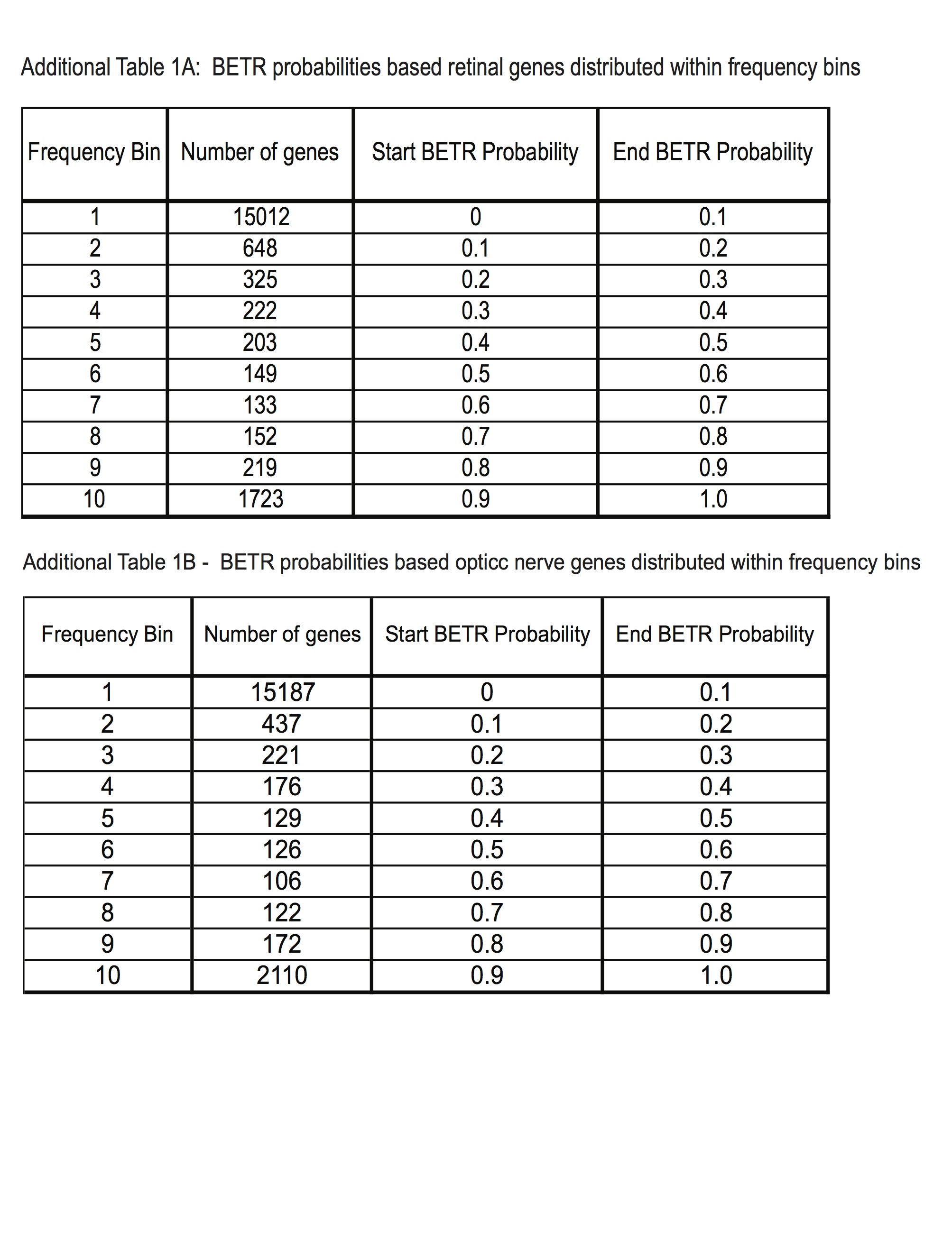

Supplement: Additional file 3: Table S1 — A, B: BETR probabilities based retinal and ON genes distributed within frequency bins. [file 1750-1326-9-14-S3.jpeg]

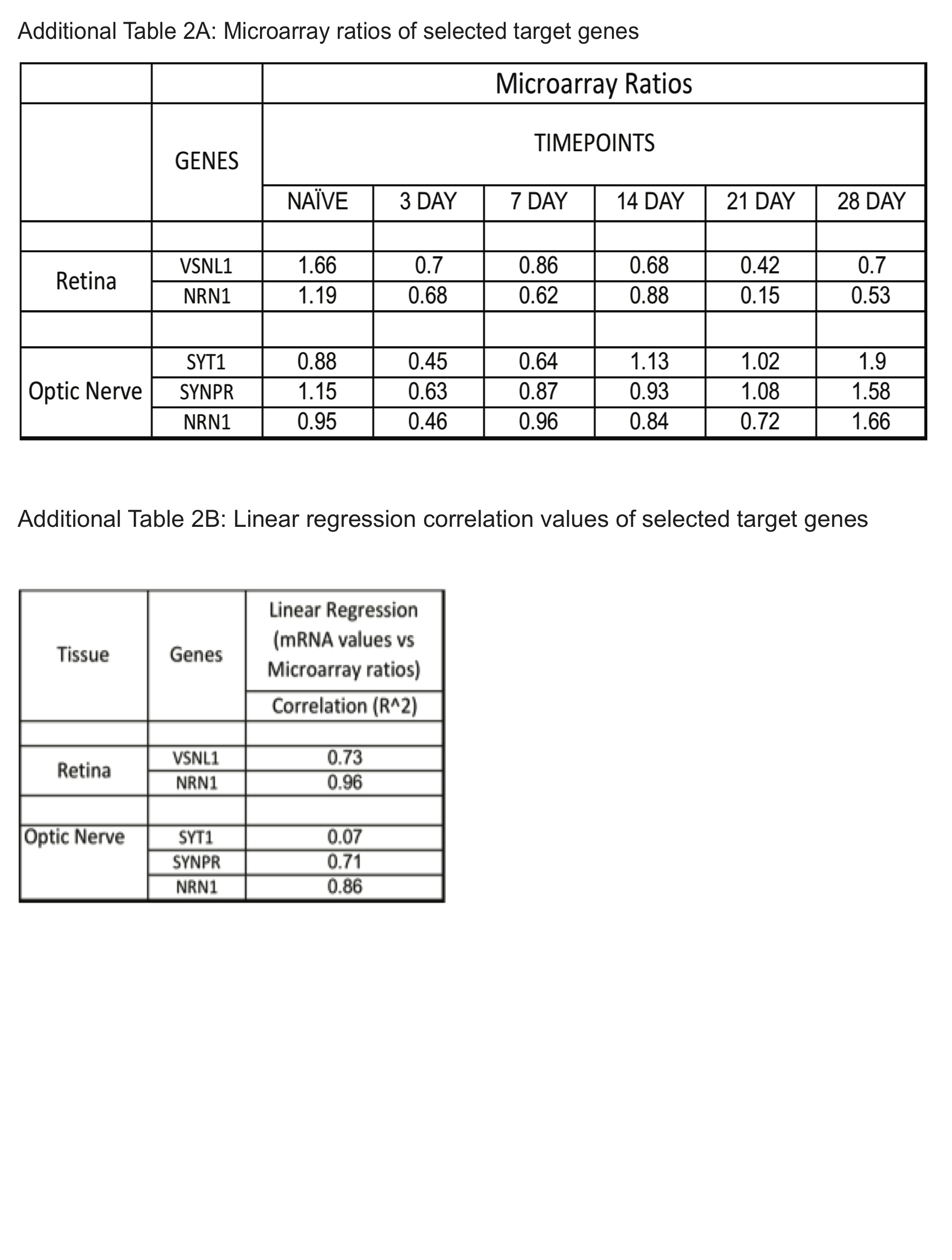

Supplement: Additional file 4: Table S2 — A, B: Microarray ratios and linear regression correlation values of selected target genes. [file 1750-1326-9-14-S4.jpeg]

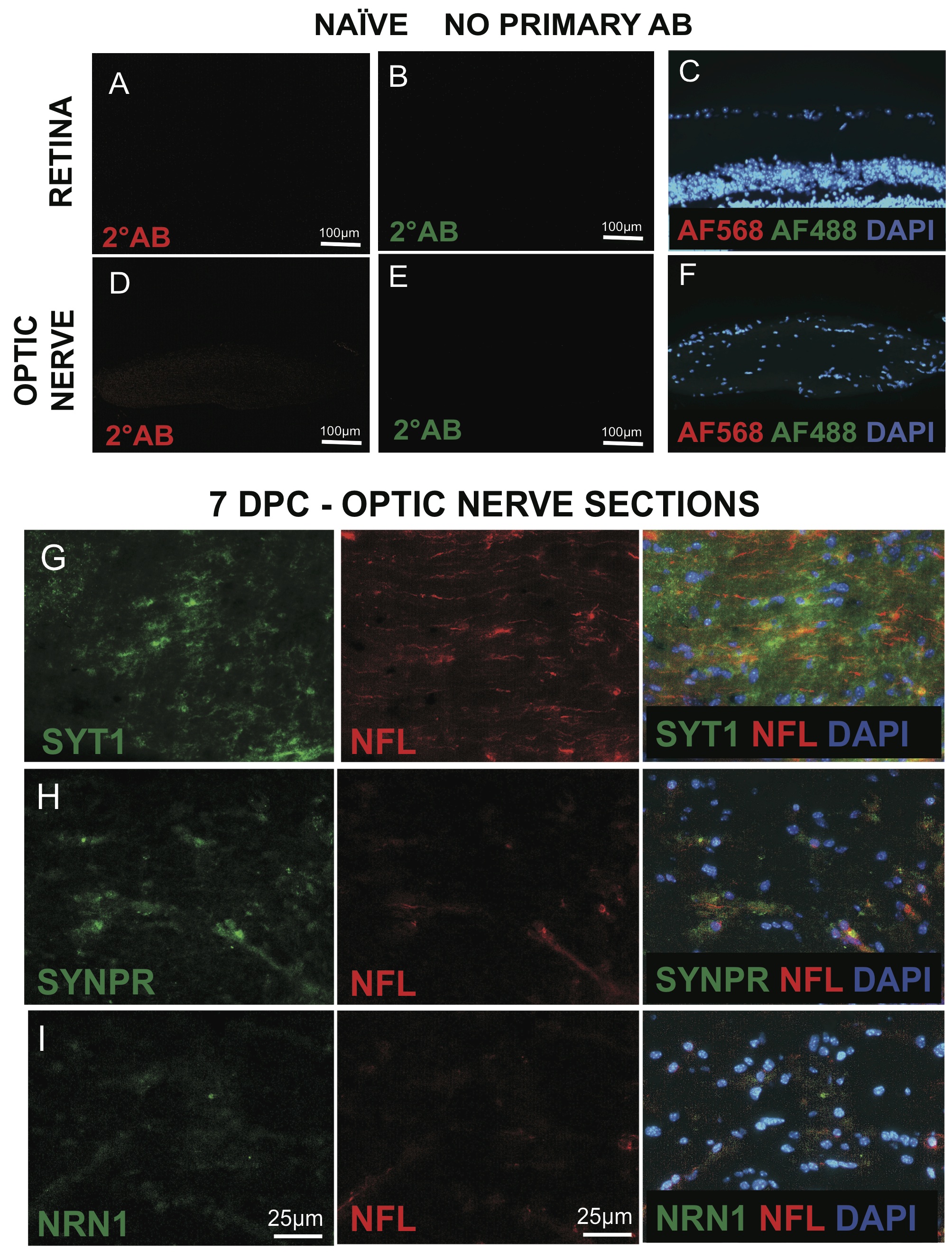

Supplement: Additional file 5: Figure S3 — A-I: Naïve control images and expression of Syt1, Synpr, Nrn1 and Nfl in the ON at 7 days post crush. [file 1750-1326-9-14-S5.jpeg]

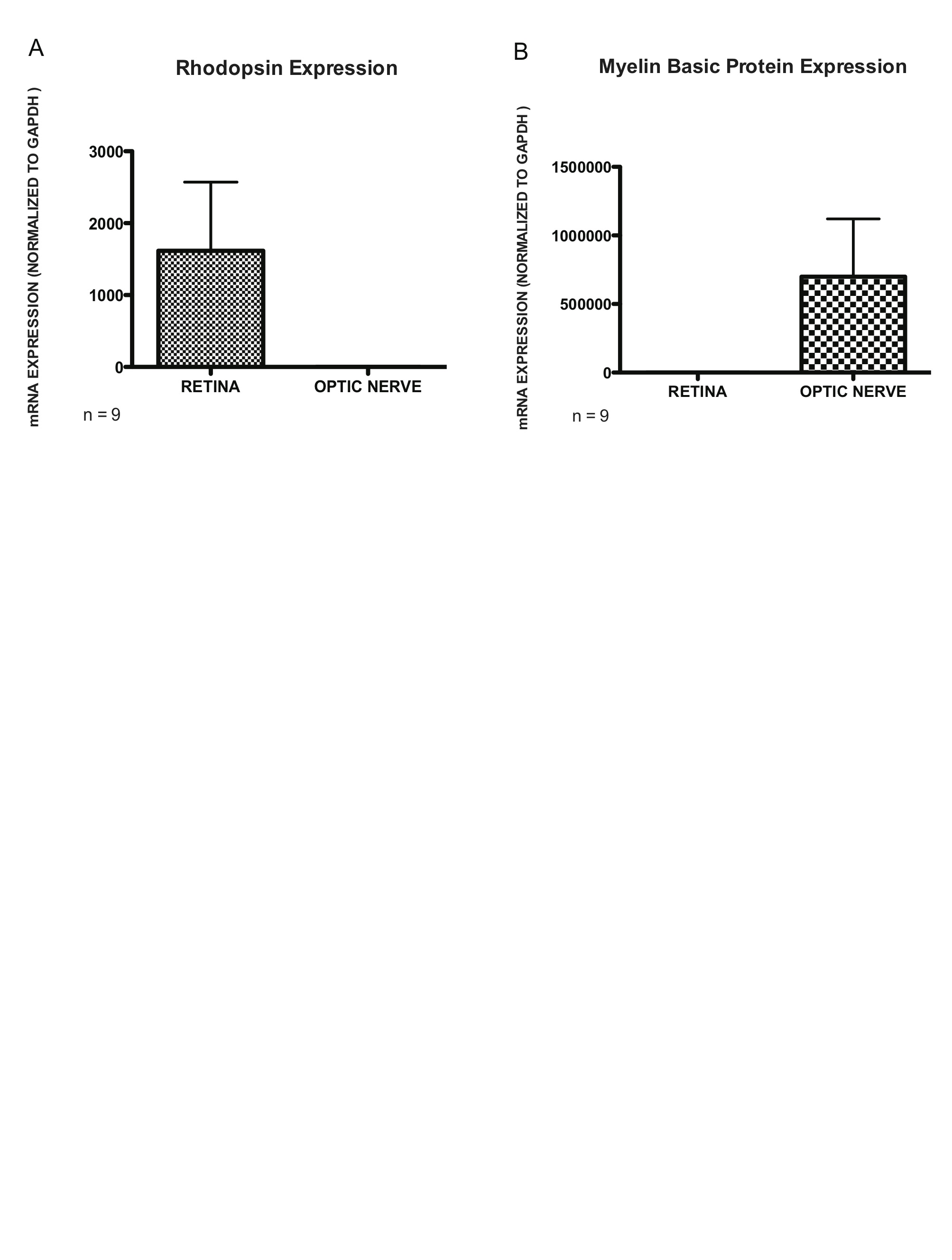

Supplement: Additional file 6: Figure S4 — A, B: Expression of tissue specific genes within normal retina and ON samples. [file 1750-1326-9-14-S6.jpeg]

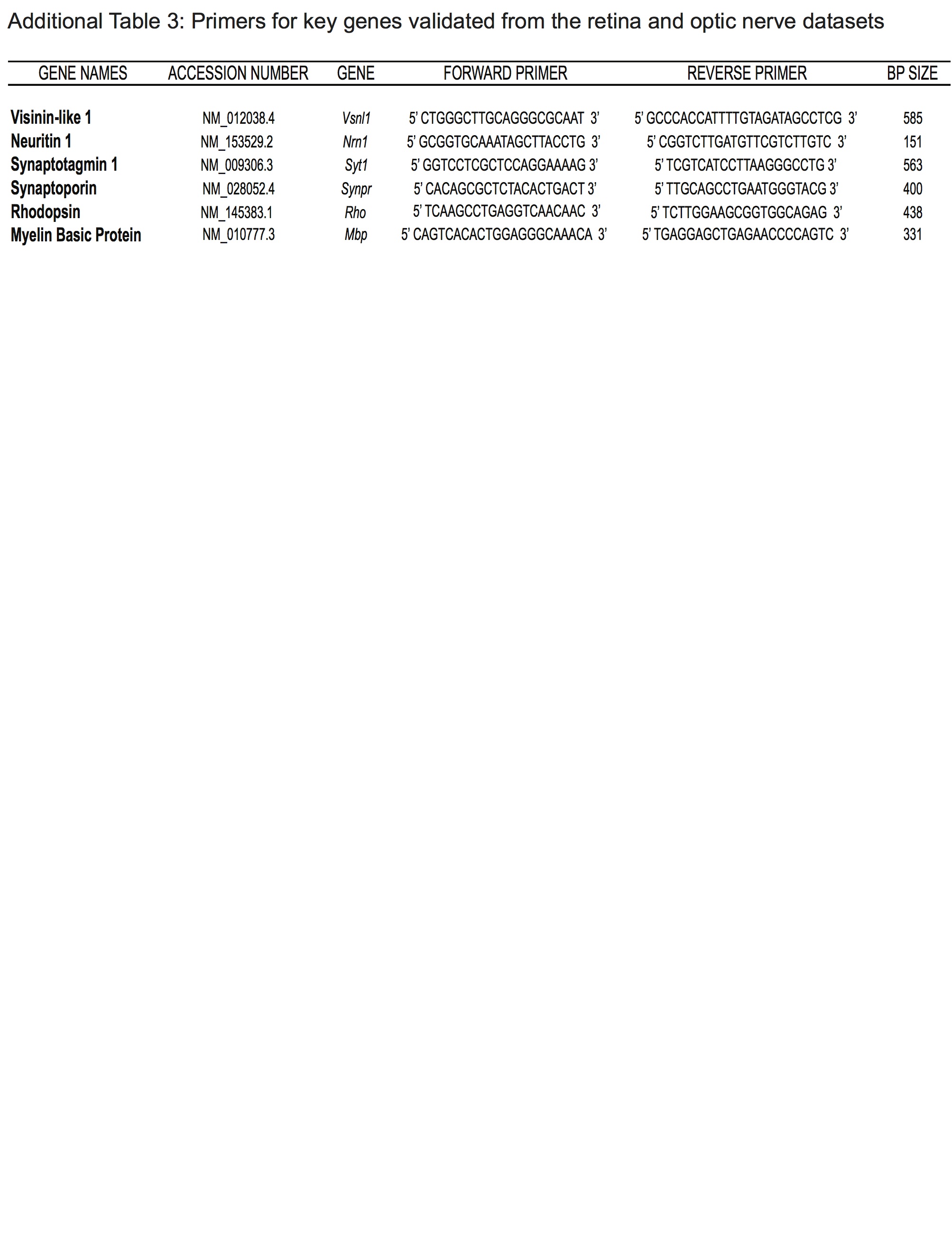

Supplement: Additional file 7: Table S3 — Primers for key genes validated from the retina and optic nerve datasets. [file 1750-1326-9-14-S7.jpeg]

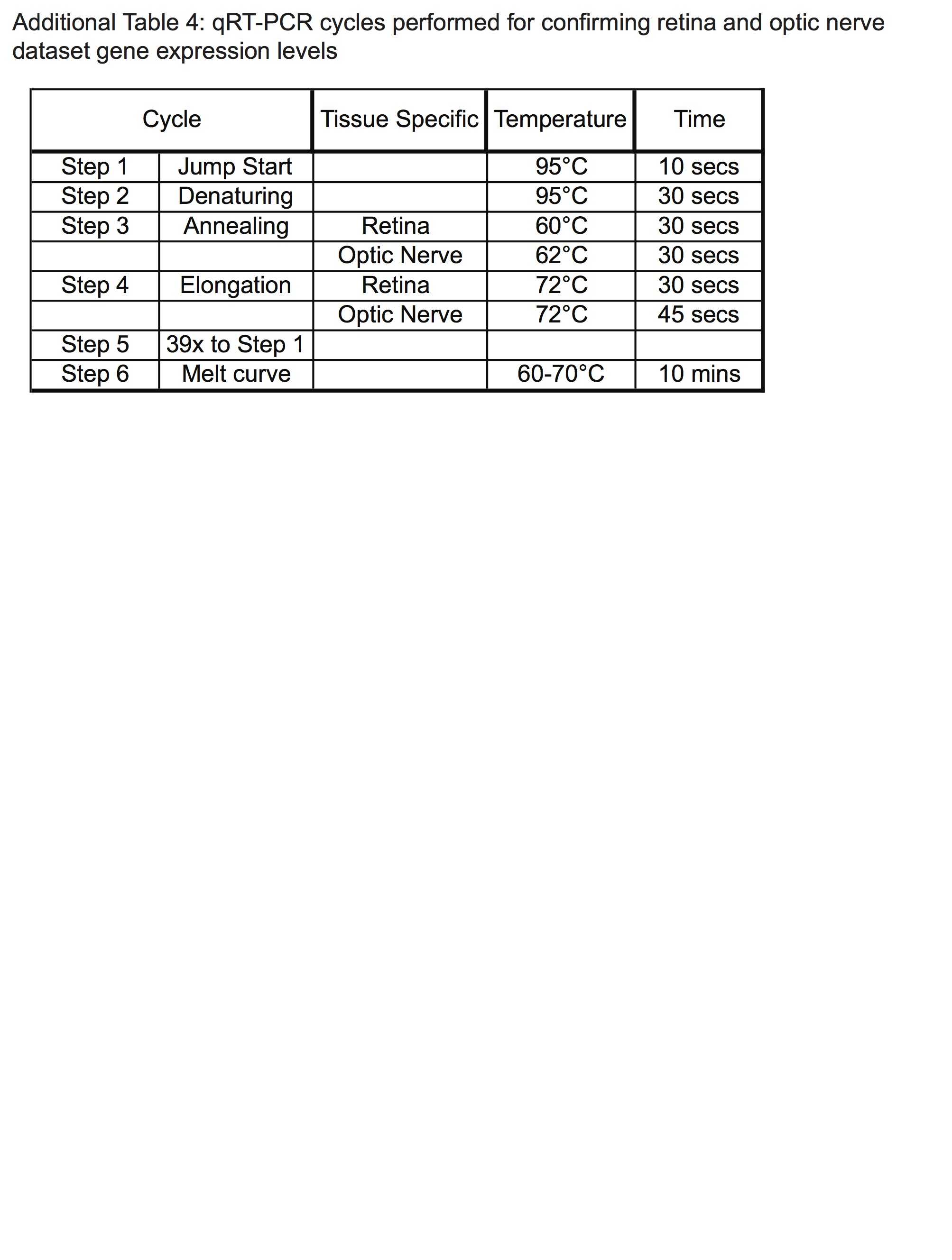

Supplement: Additional file 8: Table S4 — qRT-PCR cycles performed for confirming retina and optic nerve dataset gene expression levels. [file 1750-1326-9-14-S8.jpeg]

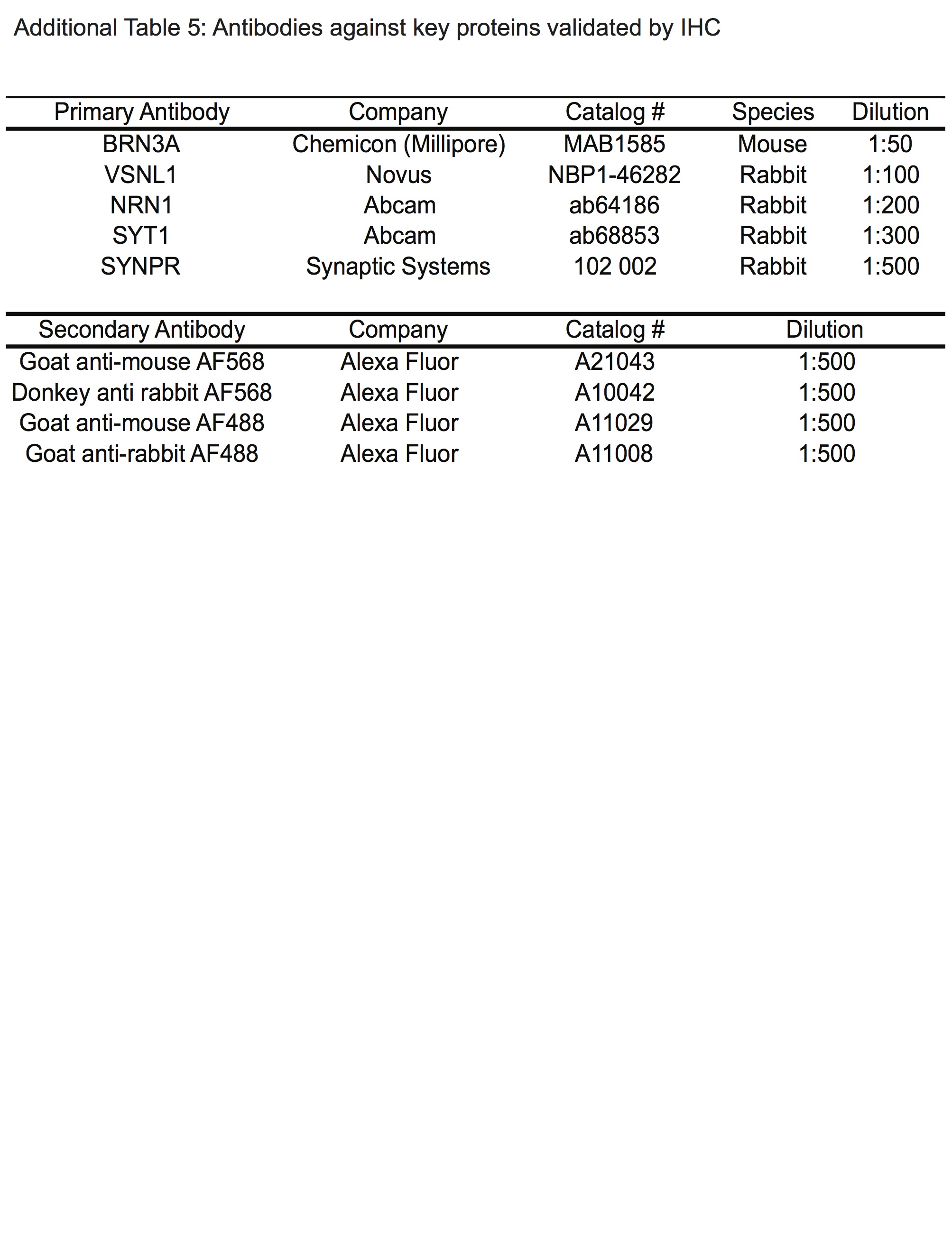

Supplement: Additional file 9: Table S5 — Antibodies against key proteins validated by IHC. [file 1750-1326-9-14-S9.jpeg]
